# Apoer2-ICD-dependent regulation of hippocampal ribosome mRNA loading

**DOI:** 10.21203/rs.3.rs-3040567/v1

**Published:** 2023-06-28

**Authors:** Catherine Wasser, Gordon C. Werthmann, Eric M Hall, Kristina Kuhbandner, Connie H Wong, Murat S Durakoglugil, Joachim Herz

**Affiliations:** UT Southwestern: The University of Texas Southwestern Medical Center; UT Southwestern: The University of Texas Southwestern Medical Center; UT Southwestern: The University of Texas Southwestern Medical Center; UT Southwestern: The University of Texas Southwestern Medical Center; UT Southwestern: The University of Texas Southwestern Medical Center; UT Southwestern: The University of Texas Southwestern Medical Center; UT Southwestern: The University of Texas Southwestern Medical Center

**Keywords:** Apoer2, Alzheimer’s, TRAP-Seq, Synaptic Homeostasis, Reelin, ApoE, Alternative Splicing

## Abstract

**Background:**

ApoE4, the most significant genetic risk factor for late-onset Alzheimer’s disease (AD), sequesters a pro-synaptogenic Reelin receptor, Apoer2, in the endosomal compartment and prevents its normal recycling. In the adult brain, Reelin potentiates excitatory synapses and thereby protects against amyloid-β toxicity. Recently, a gain-of-function mutation in Reelin that is protective against early-onset AD has been described. Alternative splicing of the Apoer2 intracellular domain (Apoer2-ICD) regulates Apoer2 signaling. Splicing of juxtamembraneous exon 16 alters the g-secretase mediated release of the Apoer2-ICD as well as synapse number and LTP, and inclusion of exon 19 ameliorates behavioral deficits in an AD mouse model. The Apoer2-ICD has also been shown to alter transcription of synaptic genes. However, the role of Apoer2 splicing for transcriptional regulation and its role in AD pathogenesis is unknown.

**Methods:**

To assess *in vivo* mRNA-primed ribosomes specifically in hippocampi transduced with Apoer2-ICD splice variants, we crossed wild-type, cKO, and Apoer2 cleavage-resistant mice to a Cre-inducible translating ribosome affinity purification (TRAP) model. This allowed us to perform RNA-Seq on ribosome-loaded mRNA harvested specifically from hippocampal cells transduced with Apoer2-ICDs.

**Results:**

Across all conditions, we observed ~ 4,700 altered ribosome-associated transcripts, several of which comprise key synaptic components such as extracellular matrix and focal adhesions with concomitant perturbation of critical signaling cascades, energy metabolism, translation, and apoptosis. We further demonstrated the ability of the Apoer2-ICD to rescue many of these altered transcripts, underscoring the importance of Apoer2 splicing in synaptic homeostasis. A variety of these altered genes have been implicated in AD, demonstrating how dysregulated Apoer2 splicing may contribute to neurodegeneration.

**Conclusions:**

Our findings demonstrate how alternative splicing of the APOE and Reelin receptor Apoer2 and release of the Apoer2-ICD regulates numerous ribosome-associated transcripts in mouse hippocampi *in vivo*. These transcripts comprise a wide range of functions, and alterations in these transcripts suggest a mechanistic basis for the synaptic deficits seen in Apoer2 mutant mice and AD patients. Our findings, together with the recently reported AD-protective effects of a Reelin gain-of-function mutation in the presence of an early-onset AD mutation in Presenilin-1, implicate the Reelin/Apoer2 pathway as a target for AD therapeutics.

## Background

Apolipoprotein E receptor 2 (Apoer2) and the very-low density lipoprotein receptor (Vldlr) are single-pass membrane proteins that act in concert with their ligand Reelin to orchestrate essential processes directing neurons to their final positions during development (Forster et al., 2010). They are later repurposed as homeostatic regulators at the synapse where they are involved in synaptic formation, maturation, and strength (Niu et al., 2008; Qiu and Weeber, 2007). In humans, there are three isoforms of ApoE (ε2, ε3, ε4) which differ from each other by one or two amino acids. These coding polymorphisms are associated with a drastically different age of onset of late-onset Alzheimer’s disease (LOAD), whereby Apoe2 confers protection against and ApoE4 is the leading genetic risk factor for LOAD (Corder et al., 1993; Strittmatter and Roses, 1996).

The Apoer2 ligand, Reelin, is implicated in several neuropsychiatric and neurodegenerative diseases, such as autism, schizophrenia, bipolar disorder, major depression, and AD (reviewed in (Caruncho et al., 2004; Costa et al., 2001; D’Arcangelo, 2006; Folsom and Fatemi, 2013; Knuesel, 2010; Lakatosova and Ostatnikova, 2012; Lane-Donovan et al., 2015)). Functional Reelin expression is altered in AD (Botella-Lopez et al., 2006; Chin et al., 2007; Herring et al., 2012), and its protective effects are antagonized by Aβ toxicity (Lane-Donovan et al., 2015; Pujadas et al., 2014). Gene polymorphisms and altered splice variants of the ApoE/Reelin receptors, Apoer2 (Helbecque et al., 2009; Hinrich et al., 2016a) and Vldlr (Yamanaka et al., 1998) have been detected in AD patients. Most importantly, a gain-of-function mutation in Reelin has recently been reported to be protective against an aggressive, heritable form of early-onset AD (Lopera et al., 2023). The protective effect of Reelin gain-of-function mutations combined with the disease-associated effect of Apoer2 loss-of-function provides strong genetic evidence for this pathway at the core of AD pathophysiology.

How Apoer2 signaling regulates neuronal homeostasis is unknown. Alternative splicing of Apoer2 plays a critical role in synapse function and number (Beffert et al., 2005; Dumanis et al., 2011; Forster et al., 2010; Niu et al., 2008; Qiu and Weeber, 2007; Reddy et al., 2011; Strasser et al., 2004; Sun and Soutar, 1999; Wasser et al., 2014), indicating a physiological need for functionally diverse forms of the receptor. Much like other receptors (i.e. LRP1 and Notch), Apoer2 undergoes sequential cleavage (Herz and Bock, 2002; May et al., 2003; Shah et al., 2005; Zheng and Koo, 2006), releasing cleavage fragments on extracellular and intracellular sides of the membrane (Apoer2-ECD and -ICD, respectively). The Apoer2-ICD released by γ-secretase is reported to translocate to the nucleus and alter gene transcription similarly to the Lrp1-ICD (Balmaceda et al., 2014; Koch et al., 2002; May et al., 2003; May et al., 2002; Telese et al., 2015, Zurhove et al., 2008).

When splicing skips exon 16, Apoer2 lacks the O-linked sugar domain and is resistant to the initial proteolytic cleavage by extracellular metalloproteases, preventing the sequential release of the Apoer2-ECD and -ICD (Wasser et al., 2014). Mice expressing this cleavage-resistant Apoer2 (Apoer2^Δ16^) in place of the endogenous receptor (Apoer2 knockin, KI) lack release of the Apoer2-ECD/ICD and have more Apoer2, more synapses, and reduced synaptic function (Wasser et al., 2014). A second alternatively spliced exon (19 in mice, 18 in humans) encodes a proline-rich cytosolic domain, which is differentially spliced in an activity-dependent manner (Beffert et al., 2005; Stockinger et al., 2000). The inclusion of exon 19 is required for Reelin-mediated LTP enhancement through binding PSD-95 (Beffert et al., 2005).

A human study of postmortem AD brains revealed increased exclusion of the proline-rich domain of Apoer2 (Hinrich et al., 2016b). The same splicing defect was observed in the brains of an AD mouse model, which have amyloid plaque deposition detectable at 3 months of age (Hinrich et al., 2016b). Delivery of antisense oligonucleotides that block exclusion of exon 19 prevented the cognitive deficits in this model (Hinrich et al., 2016b). While the role of this proline rich domain is unclear, exclusion of exon 19 in the cleavage-resistant Apoer2^Δ16^ KI mice (Apoer2^Δ16Δ19^) enhances the synaptogenic effect and further exacerbates the synaptic dysfunction compared to those with the proline-rich domain (Apoer2^Δ16+19^). This suggests that inclusion of exon 19 can attenuate the loss of Apoer2 cleavage independent of transcriptional regulation which relies on cleavage of the Apoer2-ICD (Telese et al., 2015; Wasser et al., 2014).

To probe for the role of Apoer2-ICD splice variants, we sought to uncover whether the Apoer2-ICD is necessary and sufficient to regulate ribosome loading of key neuronal transcripts. This required a multi-model approach with three Apoer2 mouse lines – each lacking the release of the Apoer2-ICD and crossed to the Rosa26-TRAP (translating-ribosome affinity purification) mouse, which harbors a Cre-inducible GFP-tagged ribosomal subunit (Heiman et al., 2014; Heiman et al., 2008. Two of these lines express a cleavage-resistant Apoer2, Apoer2^Δ16±19^ while the third is a Cre-inducible conditional knockout Apoer2 line, Apoer2^cKO^. Upon lentiviral delivery of Cre with or without the Apoer2-ICD[± 19], we assessed the role of the Apoer2-ICD in regulating neuronal ribosome-associated transcripts and the impact of alternative splicing on this regulation. Importantly, this method allowed us to isolate and sequence mRNA-primed ribosomes specifically from hippocampal cells *in vivo* which expressed exogenous soluble Apoer2-ICD, therefore ensuring changes to ribosome-bound mRNA were specifically due to re-introduction of the Apoer2-ICD. When comparing our sequencing datasets to AD genetic risk loci, the ribosome association of 34 AD risk transcripts are differentially regulated. These findings are consistent with new genetic linkages with the Reelin signaling pathway in AD pathogenesis (Bracher-Smith et al., 2022, Lopera et al., 2023), pointing to a likely underlying mechanism of Apoer2 regulation of the Reelin signaling pathway in AD.

## Methods

### Experimental Models and Subject details

All mice were housed under a 12:12 light:dark cycle and fed a normal chow diet. All animals were euthanized by inhalation of isoflurane followed by decapitation according to strict regulations set by the National Institutes of Health Guide for the Care and Use of Laboratory Animals and the UT Southwestern Animal Care and Use Committee. All mice were maintained on a wild-type SV129 and C56BL/6J mixed background. The Apoer2^Δ16±19^ mouse lines have been previously described (Wasser et al., 2014). The conditional Apoer2 knockout was created by flanking exons 1 and 2 of the *Lrp8* gene with LoxP sites. TRAP mice expressing a GFP-tagged ribosomal subunit (L10a:GFP) after Cre-induced excision of an upstream floxed stop codon were purchased from Jackson Labs (Rosa26fsTRAP, Jax no: 022367).

### Constructs

All sequences used are listed in Table S11. For the luciferase assay, 2.6-kb of the promoter regions of either human *Reln* or mouse *Lrp8* were cloned into the multiple cloning site of the Gaussia luciferase and secreted alkaline phosphatase reporter cloning vector (GeneCopoeia; pEZX-GA01; Cat#ZX103). The Apoer2-ICD expression plasmids were modified from (Beffert et al., 2005) to include an N-terminal 3x-FLAG with or without a C-terminal VP16. For the TRAP constructs, the 3x-FLAG and Apoer2-ICD sequences were cloned into the pLVX-IRES-ZsGreen1 Vector (Clontech Cat#632187) and the ZsGreen sequence was replaced with the Cre-recombinase sequence.

### Luciferase assay

HEK-293T or SH-SY5Y cells were co-transfected with dual reporter construct containing a CMV-driven secreted alkaline phosphatase (SEAP) and a Reelin promoter-driven secreted Gaussia luciferase (GLuc) reporter construct (GeneCopoeia, cat. no:pEZX-GA01) and either GFP or an Apoer2-ICD (Apoer2-ICD:VP16 construct (± exon 19, Apoer2-ICD[+ 19/Δ19] ± VP16). After 24 hours, the media was collected from transfected cells and the luciferase and control SEAP intensity was quantified. Luciferase signal was normalized to the SEAP signal for each well, then all values were normalized to media from cells transfected with an empty reporter construct and GFP. (3–5 wells per condition, 2 parallel measurements, at least 2 independent experiments).

### Lentiviral production

HEK-293T cells at 70–80% confluency in 10-cm plates were co-transfected with 3ug lentiviral construct, packaging, and envelope plasmids (ratio – 4:3:1) using Fugene6 transfection reagent following the manufacturers’ instructions. Briefly, recombinant lentiviruses were produced by co-transfecting the cells with packaging (psPAX2; 2.25 μg) and the envelope vectors (pMD2.G; 0.75 μg) along with each lentiviral transfer vector (3 μg). Eight hours later, the cells were washed and fed with fresh culture medium containing 1mM sodium butyrate. The supernatant containing lentivirus particles was collected 48h after transfection followed by filtering through a 0.45μm filter and then concentrated to < 100ul with a 10/30 kDa Amicon ultrafiltration filter. The concentrated lentivirus was layered over 10% sucrose (50mM Tris HCl pH 7.4, 100mM NaCl, 0.5mM EDTA, 10% sucrose) at a 4:1 ratio, then centrifuged at 14000 × g for 3 hours at 4°C. The subsequent pellet was resuspended in 1/100th of the original lentiviral media collected (Jiang et al., 2015).

### Lentivirus injection

Under continuous isoflurane anesthesia, 2–3 month old mice were stereotaxically injected (coordinates were AP: −2.2, ML: ±1.3, DV, −1.3) with concentrated lentivirus bilaterally to the CA1 hippocampal region with one of the three lentiviral constructs to express Cre only or both Cre and the Apoer2-ICD[± 19] (empty-IRES-Cre and Apoer2-ICD[±]-IRES-Cre). A total of 48 mice were injected (24 male, 24 female).

### Translating ribosome affinity purification (TRAP)

#### Affinity matrix preparation.

The matrix was prepared as described in (Heiman et al., 2014). For one hippocampus, the ratio of the components was 300ug Streptavidin MyOne T1 Dynabeads: 20μg biotinylated protein L: 50μg each of GFP antibodies 19C8 and 19F7 (100 μg total antibody). The appropriate amount of resuspended Dynabeads were washed once with 1X PBS (1:10 dilution of Phosphate-Buffered Saline (10X) from Invitrogen^™^ AM9625). Beads were then rotated end-over-end with biotinylated protein L in 1× PBS for 1 hour at room temperature and washed five times with 3% BSA (in 1X PBS). The protein L-conjugated beads were then rotated end-over-end for 1 hour with the GFP antibodies in a low-salt buffer (in RNase-free water: HEPES (pH 7.3), 20mM; KCl, 150mM; MgCl_2_, 10mM; NP-40, 1%; add immediately before use: DTT, 0.5mM; cyclohexamide, 100μg/ml). Antibody-conjugated beads were not vortexed after this step. After washing three times with the low-salt buffer, the affinity matrix was aliquoted into tubes for individual hippocampal immunoprecipitations. Note: Before all bead washing steps, tubes were kept against the magnet for at least one minute before removing solutions to prevent loss of beads.

#### Tissue isolation and homogenization.

Two months post-lentiviral injection, mice were anesthetized with isoflurane, quickly decapitated and the whole brain removed and placed on an ice-cold metal sheet. The brain was halved and placed into an icy slurry of cyclohexamide-containing dissection buffer (in RNase-free water: HEPES (pH 7.3), 2.5mM; HBSS, 1X; glucose, 35mM; NaHCO_3_, 4mM; add immediately before use: cyclohexamide, 100ug/ml). Dissections were performed on the left hemisphere while keeping the other hemisphere in the slurry, the cerebellum and cortex were quickly dissected from the first hemisphere and snap frozen in liquid nitrogen. The hippocampus was immediately homogenized in tissue-lysis buffer (1mL/25–50mg) (in RNase-free water: HEPES (pH 7.3), 20mM; KCl, 150mM; MgCl_2_, 10mM; add immediately before use: EDTA-free protease inhibitors, 1tab; DTT, 0.5mM; cyclohexamide, 100ug/ml; rRNasin, 10ul/ml; HEPES (pH 7.3), 20mM; SUPERase·In, 10ul/ml) with a 1-mL glass dounce homogenizer on ice followed by 10 strokes through a 23-gauge syringe. Lysates from the left hemisphere were incubated on ice while dissecting and homogenizing the right hippocampi. Lysates were centrifuged for 10 minutes at 2,000 × g at 4°C to remove nuclei (S2), and the supernatant was transferred to a fresh tube. A 1/9 volume of 10% NP-40 was added (1% final), followed by gentle inversion to mix and a brief pulse spin to prevent lysate loss. A 1/9 volume of 300mM DHPC (30mM final, prepared fresh each week in RNase-free water), followed by inversion to mix and incubated on ice for 5 minutes before centrifuging for 20 minutes at 20,000 × g at 4°C to remove mitochondria (S20). A small aliquot (~ 5%) was kept and stored at 4°C until after final RNA purification. The remaining supernatant was transferred to a fresh tube for immunopurification.

#### Immunopurification.

The appropriate amount of freshly washed and resuspended affinity matrix (See Affinity matrix preparation section) was added to each lysate and incubated overnight (~ 18 hours) at 4°C with gentle end-over-end mixing. The next day, always keeping the tubes on ice, the beads were washed to reduce non-specific binding prior to RNA purification as described in (Heiman et al., 2014). Tubes were pulse-centrifuged and beads were allowed to collect for at least one minute before each washing step. Briefly, tubes were quickly pulse-centrifuged then placed against the magnetic rack surrounded by ice. The GFP:L10-depleted lysate was removed and stored at −80°C. The beads bound to the RNA-GFP:L10 complexes were washed 4 times by resuspending beads by pipetting with 1mL of high-salt buffer (in RNase-free water: HEPES (pH 7.3), 20mM; KCl, 350mM; MgCl_2_, 10mM; NP-40, 1%; add immediately before use: DTT, 0.5mM; cyclohexamide, 100ug/ml). Of note, during each wash, beads were pipetted at least 3 more times after visible resuspension and bubbles were avoided. After removing the fourth wash, beads were removed from the magnet and warmed to room temperature before RNA purification.

RNA purification RNA was isolated from the GFP:L10-bound affinity matrix with the Absolutely RNA Nanoprep kit (Agilent, see Materials section) at room temperature. Briefly, 100μl of Nanoprep lysis buffer (with fresh ß-ME) was added to the beads. Tubes were vortexed and incubated for 10 minutes at room temperature. The tubes were then placed back on the magnet and the RNA-containing lysate was removed for purification according to the manufacturer’s protocol with the following exception. Purified RNA was resuspended in 10uL of RNase-free water instead of the Elution buffer provided with the kit. IP RNA was aliquoted before freezing at −80°C. This elution buffer interfered with the accurate assessment of quality and approximate quantity of RNA by the 2100 Bioanalyzer with the Total RNA Pico chip (performed by the UTSW Genomics Sequencing Core). We obtained pico- to nanogram amounts of IP-RNA.

### RNA amplification/ RNA-sequencing

RNA amplification RNA amplification was performed as described in (Morris et al., 2011) with the exception that we used Superscript IV instead of Superscript III. aRNA concentration was estimated with the Agilent PicoChip before submitting aRNA for library preparation and RNA-sequencing. For RNA-sequencing, we needed a minimum of 50ng of RNA, as we wanted to sequence samples separately, we performed a pilot experiment where we amplified 100pg pooled IP RNA from a subset of our IP RNA. This pilot experiment confirmed that we could successfully sequence amplified IP RNA. We then amplified 100pg of our highest quality IP RNA from each genotype and then assessed the quality and rough concentration before submitting 48 amplified RNA samples for library preparation and RNA-sequencing (6–9 mice/genotype, with at least one male or female represented per condition). Libraries were prepared with the Illumina TruSeq Stranded mRNA kit and run on NextSeq sequencing SE-75 (all across 3 flow cells) by the UTSW Genomics Sequencing Core.

### Bioinformatics

**Figure 1:**
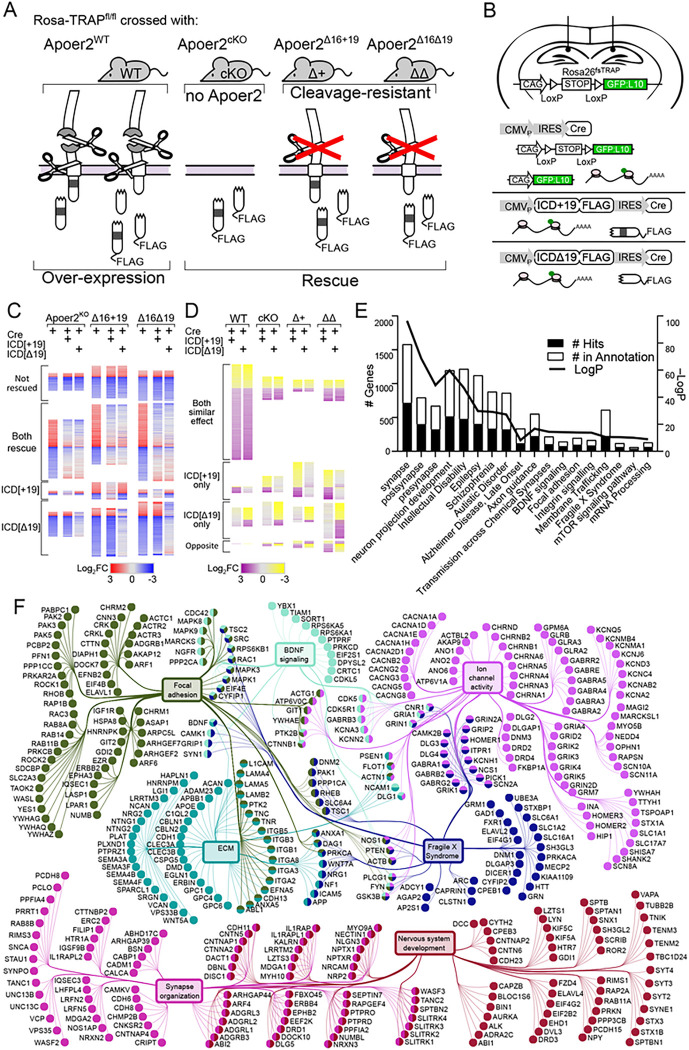
Apoer2-ICD regulation of ribosome-associated transcripts *in vivo*. (**A**) Rosa^26fs-TRAP^ mice crossed with the Apoer2^WT^, conditional KO, and cleavage-resistant Apoer2 transgenic mice (Apoer2^Δ16±19^) were injected with lentiviruses expressing Cre resulting in a GFP-tagged ribosomal subunit (L10a:GFP) to allow for pull-down of ribosome-bound transcripts (intrahippocampal injections; coordinates were AP: −2.2, ML: ±1.3, DV, −1.3). Using an Internal Ribosome Entry Site (IRES), Apoer2-ICD^[±19]^ was co-expressed to assess the effect of overexpression of either Apoer2-ICD in Apoer2^WT^ or rescue of effects of lack of the Apoer2-ICD (**B**). (**C-D**) Heatmaps representing the proportion of genes altered in Apoer2 transgenic mice that are either rescued or not rescued with the Apoer2-ICD*[±19]* (**C**) or altered by overexpression of either Apoer2-ICD in Apoer2^WT^ or (**D**) effects of the ICD independent of the lack of ICD-release. (**E**) Enrichment analysis of the ~4700 transcripts differentially translated across all conditions, demonstrating enrichment for synaptic compartments, neuronal processes and pathways, as well as diseases of the brain. (**F**) Gene-term network of the ClueGO/Cluepedia enrichment analysis of synaptic transcripts annotated in the SynGO database. (For each mouse line: Cre-only and Cre+Apoer2-ICD[+19]: n= 4 individual and one pooled set of 4 RNA samples, Cre+Apoer2-ICD[Δ19]: n= 4 individual RNA samples)

Reads were uploaded to the Galaxy online resource (https://usegalaxy.org/) and aligned to the mouse genome (mm10) using the STAR package (version 2.5.2b-0, (Dobin et al., 2013)) with the ENCODE annotation (M21, ENCFF871VGR, (Consortium, 2012; Luo et al., 2020)). To remove 3’UTR bias in amplified RNA reads, the reads mapping to UTRs were filtered from the aligned BAM files. Gene counts were calculated by featureCounts (version 1.6.4 + galaxy2, (Liao et al., 2014)). Differential gene expression was performed with the Deseq2 package (version 2.11.40.6 + galaxy1, (Love et al., 2014)) to quantify the differential expression within genotypes with and without either Apoer2-ICD. Each condition was also tested against the Apoer2^WT^ injected with the empty-IRES-Cre lentivirus. Significant basal differences of genotype compared to Apoer2^WT^ were defined as |log_2_FC|>0.56 and p-value < 0.05. “Rescued” transcripts were defined as having a smaller log_2_FC and an adjusted p-value < 0.05. These adjusted p-values take into account the baseline effect of genotype compared to wildtype and the effect of the ICD in the genotype, thus increasing the power of the experiment. This is calculated by squaring the sum of each p-value and dividing this by the number of p-values summed (Colombo et al., 2017). Filtering by the adjusted genotype-rescue p-values is the most relevant measure of ICD effect. Heatmaps were created with Morpheus (https://software.broadinstitute.org/morpheus/) and hierarchical clustering was performed with Euclidian distance using complete linkage. Supervenns were created with the Compare Sets Appyter (Clarke et al., 2021). Protein-protein interactions were created with STRING and Metascape (Zhou et al., 2019). These programs were also used to find functional enrichments along with ToppFun (Chen et al., 2009) and DAVID(Huang da et al., 2009). Gene families were identified with Gene Set Enrichment Analysis (GSEA) online (https://www.gsea-msigdb.org/gsea/msigdb/) (Mootha et al., 2003; Subramanian et al., 2005). The core minimal network was comprised from the following enrichment categories: synapse organization (GO:0034329: cell junction assembly; GO:0034330: cell junction organization; GO:0050807, GO:0099175: regulation of synapse/postsynapse organization), neuron development (GO:0051960: regulation of nervous system development; GO:0048666: neuron development; GO:0048699: generation of neurons; GO:0022008: neurogenesis), focal adhesion (Wikipathway, WP306; KEGG, hsa04510; GO:0005925), extracellular matrix (ECM: GO:0030198, REACTOME, R-HSA-1474244), Fragile X Syndrome (Wikipathway, WP4549), BDNF signaling (Wikipathway, WP2380), and ion channel activity (GO:0005216) (Fig. 1F). Cell type approximation (Figure S5) was performed by comparing differentially expressed ribosome-associated transcripts to a previously published single-cell RNA-Seq hippocampal dataset (Karlsson et al., 2021).

### Data analysis

Statistical analyses were performed with Graphpad Prism 8 software using one-way and two-way ANOVA with Tukey’s *post hoc* multiple comparisons test for exact multiplicity adjusted p-values between groups. All data sets were checked for normality with the D’Agostino & Pearson omnibus or KS normality test. If data was non-normal, the non-parametric Kruskal-Wallis test was performed with Dunn’s *post hoc* multiple comparisons test.

## Results

### Apoer2-ICD regulation of ribosome-associated transcripts in vivo

Apoer2 plays a critical role in synaptic function and number – both are regulated by alternative splicing (Beffert et al., 2005; Dumanis et al., 2011; Forster et al., 2010; Niu et al., 2008; Qiu and Weeber, 2007; Strasser et al., 2004; Wasser et al., 2014). This splicing regulates Apoer2 cleavage as well as synaptic plasticity and interactions (Beffert et al., 2005; Reddy et al., 2011; Wasser and Herz, 2017; Wasser et al., 2014); however, the role of splicing in Apoer2-ICD-dependent transcript regulation is unknown. Mice expressing the cleavage-resistant Apoer2 (Apoer2^Δ16^) in place of the endogenous receptor (Apoer2 knockin, KI) lack release of the Apoer2-ECD/ICD and have more Apoer2 mRNA and protein, more synapses, and reduced synaptic function (Wasser et al., 2014). Exclusion of exon 19 in the cleavage-resistant Apoer2^Δ16^ KI mice (Apoer2^Δ16Δ19^) further exacerbates the synaptic dysfunction and synaptogenesis resulting from the loss of Apoer2 cleavage (Apoer2^Δ16^), suggesting that the proline-rich domain does impart a synaptic effect independent of the release of the ICD (Wasser et al., 2014).

To determine how the Apoer2-ICD and its splicing regulate synapses, we crossed Rosa26-TRAP mice, which express a Cre-inducible GFP-tagged ribosomal subunit (L10a:GFP), to Apoer2 mutant mice which lack all or part of the Apoer2-ICD: a conditional Apoer2 knockout (Apoer2^cKO^) and two cleavage-resistant Apoer2 lines (Apoer2^Δ16^) either with the proline-rich domain (Apoer2^Δ16+19^) or without it (Apoer2^Δ16Δ19^) (Heiman et al., 2014; Heiman et al., 2008) (Fig. 1A). We then delivered the Apoer2-ICD with or without exon 19 (Apoer2-ICD[± 19]-IRES-Cre) to the hippocampi of these mice via lentiviral injection to demonstrate the sufficiency of the Apoer2-ICD to alter the expression of key synaptic transcripts (Fig. 1B–C). We next performed next generation RNA-Seq specifically on hippocampal cells infected with Cre-expressing lentivirus, allowing us to precisely identify transcripts affected by the Apoer2-ICD within the complex physiology of the living brain.

We utilized this rich RNA-Seq dataset to answer key questions regarding the function of Apoer2 splicing on hippocampal mRNA primed ribosomes. First, we compared the basal effect of the loss of nuclear Apoer2-ICD transcriptional regulation by comparing the ribosome-associated transcripts from the hippocampi of Apoer2 KI/cKO to the wild-type Apoer2 injected with lentivirus expressing only Cre. We then identified whether the Apoer2-ICD variants could rescue these effects. Afterwards, we looked for the effects imparted by the expression of either Apoer2-ICD variants independent of genotype. To ensure the difference between Apoer2-ICD with and without exon 19 was not due to the inability of Apoer2-ICD[Δ19] to act as a transcriptional regulator, we tested the binding of our Apoer2-ICD constructs to the *Reln* (a known Apoer2-ICD-regulated gene) promotor using a *Gaussia* luciferase reporter construct driven by 2.5 kb of the *Reln* promoter (see methods). As the cleavage-resistant Apoer2 mice have elevated Apoer2 transcription and translation, we also evaluated whether these Apoer2-ICD splice variants bind the promoter of Apoer2 (*Lrp8* promoter) (Figure S1A-C). We found no difference between the effects of Apoer2-ICD[+ 19] and Apoer2-ICD[Δ19] on luciferase expression controlled by *Reln* and *Lrp8* promotors (Figure S1D-G). This suggests that the Apoer2-ICD can regulate the transcription of at least a subset of its target genes independent of exon 19.

Across all conditions, the ribosome association of ~ 4,700 transcripts were altered (Fig. 1C, D, Table S1–4). Approximately half of the altered transcripts were basal differences between the Apoer2 cKO/KI and the wild-type when injected with lentivirus expressing only Cre (Fig. 1C). In addition, the majority of ribosome-associated mRNAs were restored when Apoer2-ICD[± 19]-IRES-Cre was injected and a smaller portion were either not rescued or rescued by only one of the Apoer2-ICD splice variants (Fig. 1C). The other half of the overall altered transcripts were a result of genotype-independent effects of lentiviral expression of the Apoer2-ICD in the wild-type or Apoer2 cKO/KI mice (Fig. 1D). Here we find that in the Apoer2^WT^, the majority of transcripts are similarly regulated by both Apoer2-ICD splice variants, with a smaller portion regulated by only one of the Apoer2-ICD splice variants; however, across the Apoer2 cKO/KI mice the effects of the Apoer2-ICD variants are more diverse (Fig. 1D).

The overall dataset was highly enriched for synaptic compartments and neuronal processes/pathways as well as diseases of the brain by ToppFun enrichment analysis (https://toppgene.cchmc.org/enrichment.jsp) (Chen et al., 2009) (Fig. 1E). To further elucidate the effect of the Apoer2-ICD on synaptic gene regulation, we identified specific transcripts with known synaptic localization and/or function using the SynGo database (Koopmans et al., 2019) which contains manual curations from numerous published studies. Of the 4,658 altered transcripts, 610 were annotated in the database with synaptic localization and/or function. We focused on those 610 synaptic genes and created a functional enrichment network using the ClueGO (Bindea et al., 2009) with Cluepedia (Bindea et al., 2013) applications in the Cytoscape App (Lotia et al., 2013). This workflow linked ~ 443 of these synaptic genes to six core clusters: synapse organization, neuron development, focal adhesion, extracellular matrix (ECM), Fragile X Syndrome, BDNF signaling, and ion channel activity (Fig. 1F). In addition, we have also manually gathered all the transcripts annotated under these categories and expanded the network to include those not annotated in the SynGo database, resulting in a total of 1,301 transcripts. The residual 208 SynGO synaptic transcripts not included in the minimal synaptic network in Fig. 1F are enriched for neurotransmitter transport, synaptic transmission, synaptic vesicle cycle and membrane trafficking. The 3,146 non-SynGO transcripts not included in the minimal Apoer2-ICD network were enriched for mRNA processing as well as oxidoreductase activity, chromatin modification, cell cycle and metabolism of lipids.

### Basal ribosome loaded mRNA differences in Apoer2^cKO^ and cleavage-deficient Apoer2^D16^ mutant hippocampus

**Figure 2:**
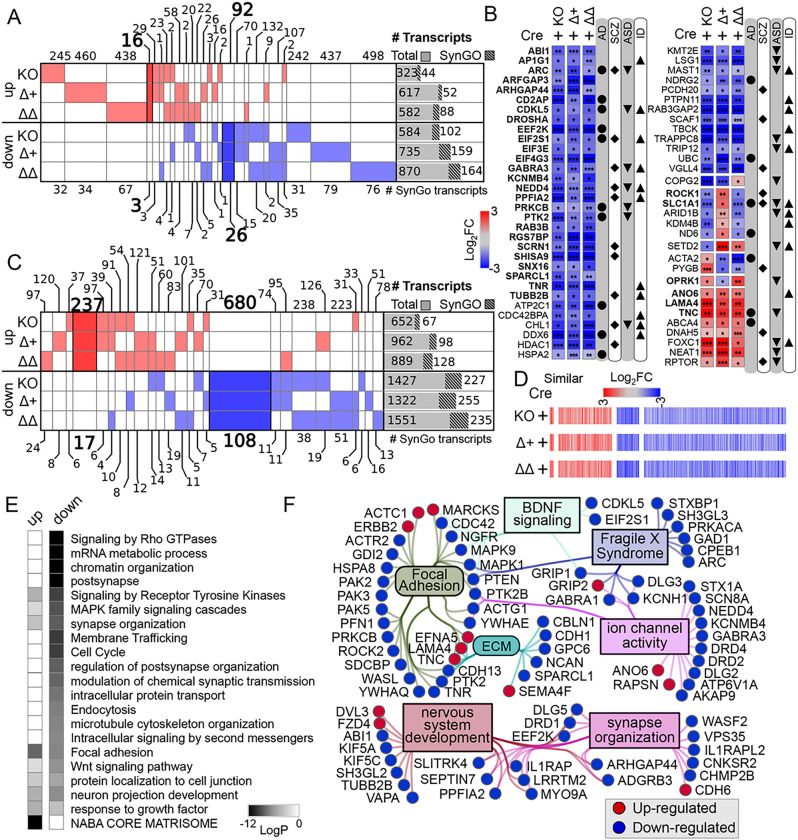
Basal translatome differences in Apoer2^cKO^ and cleavage-deficient hippocampi. (**A-B**) Supervenn and heatmap depicting the overlap of transcripts significantly altered in Apoer2 transgenic mice compared to Apoer2^WT^. (**A**) Translatome overlap of transcripts significantly (p-value<0.05, |log_2_FC|> 0.56) altered in the Apoer2 KI/cKO genotypes compared to Apoer2^WT^. (**B**) Heatmap of the 53 ribosome-associated SynGO (bolded) and disease-related transcripts (symbols) significantly altered in all three Apoer2 KI/cKO compared to Apoer2^WT^. (**C**) Expanded translatome overlap of the ~3,000 transcripts altered in the Apoer2 KI/cKO genotypes compared to Apoer2^WT^ (|log_2_FC|>0.56, p-value<0.05 in at least one). (**D**) Heatmap demonstrating the log_2_FC of expanded basal translatome in Apoer2^cKO^ and cleavage-deficient hippocampi. (**E**) Enrichment analysis of the transcripts in Panel D. (**F**) Diagram depicting the SynGO transcripts differentially-translated in the Apoer2 KI/cKO (Panel D) compared to Apoer2^WT^ (up, red circles; down, blue circles) in the network from Figure 1F. AD, Alzheimer’s disease; SCZ, schizophrenia; ASD, autism spectrum disorders; ID, intellectual disability.

When we compared the transcripts affected by complete loss of the protein (Apoer2^cKO^ with IRES-Cre) or lack of the release of the ICD (Apoer2^Δ16±19^ with IRES-Cre), there were a total of 2,953 differentially regulated ribosome-associated transcripts across all Apoer2 KI/cKO genotypes compared to Apoer2^WT^ (Figs. 1C, 2A, S3A, Table S2–4); however, only 125 (4%) of these are significantly altered in all three Apoer2^cKO^ and cleavage-deficient genotypes (Figs. 1C, 2A–B), which outlines the effects of the Apoer2-ICD on ribosomal mRNA loading. Half of these transcripts were either SynGO or known disease-associated transcripts. Of these, there are 16 up-regulated transcripts with 4 involved in the PI3K-AKT signaling pathway (COL1A2, RPTOR, LAMA4, TNC), and 92 down-regulated transcripts with 5 known to modulate neurotransmission (ARC, ARHGAP44, CDKL5, TNR, TUBB2B) and 6 involved in the synaptic vesicle cycle (PPFIA2, ABI1, AP1G1, PRKCB, RAB3B, SCRN1). Of the transcripts significantly altered in all three genotypes in opposing directions, 11 transcripts were similarly regulated in the Apoer2^cKO^ and Apoer2 Δ16Δ19 with opposite effects of Apoer2^Δ16+19^ (up, OPRK1, RCC2; down, ROCK1, SLC1A1, ARID1B, BRWD1, DSP, KDM4B, ND6, RALGAPA2, ZNF644), 4 were common between Apoer2^Δ16+19^ and Apoer2^Δ16Δ19^ with opposite effect in Apoer2^cKO^ (ACTA2, SETD2, NCOA2), and 2 were common between Apoer2^cKO^ and Apoer2^Δ16+19^ with opposite effect in Apoer2 ^Δ16Δ19^ (up, RASAL2; down, COPG2) (Fig. 2B). This demonstrates the ability of exon 19 splicing to affect synaptic homeostasis in the absence of its normal g-secretase mediated cleavage.

As all Apoer2 KI/cKO mice lack the release of the Apoer2-ICD, we expected to find more than just 4.2% of the differentially regulated transcripts in common between them when compared to Apoer2^WT^. To find similar regulatory modules within these > 2000 transcripts only, we reduced our criteria within these genes to |log_2_FC| > 0.5 (Fig. 2C–D). With this reduced criterion, we identified 237 up- and 680 down-regulated transcripts in all three Apoer2 KI/cKO (605 total, Table S5). Eighty-one of these are annotated in GSEA (Mootha et al., 2003; Subramanian et al., 2005) as transcription factors (Figure S2). Overall, these similarly regulated transcripts can be grouped into 20 functional enrichments across the three Apoer2 KI/cKO lines (Fig. 2E, Table S5). Focal adhesion transcripts were both up- and down-regulated. The other top enrichments for up-regulated transcripts were response to growth factor, neuron projection development, and matrisome. The top enrichments for down-regulation were post-synaptic transcripts, Signaling by Rho GTPases, and mRNA metabolic process (Fig. 2E, Table S5).

There were 17 up- and 108 down-regulated synaptic ribosome-associated transcripts with similar differential regulation in the Apoer2 KI/cKO lines compared to wild-type controls, suggesting potential key transcripts that are regulated by the Apoer2-ICD. Eighty-five of these transcripts were in our minimal synaptic network from Fig. 1F with 13 transcripts up- and 72 down-regulated (Fig. 2F). Of the other SynGO annotated transcripts not in the network were 4 up- and 36 down-regulated.

Only 416 transcripts were not shared between at least two of the genotypes. Of the 416 differentially changed in only one genotype, Apoer2^cKO^ has 37 up- and 33 down- regulated (up-regulation in neuron death: APOE, CASP3, MAPK8, TFAP2B), Apoer2 ^Δ16+19^ has 120 up- and 51 down- regulated, and Apoer2 ^Δ16Δ19^ has 97 up- and 78 down-regulated. The differential regulation of these synaptic ribosome-associated transcripts could provide insight into the differential phenotypes observed in these mouse lines.

### Apoer2-ICD regulation in the Apoer2^WT^

**Figure 3:**
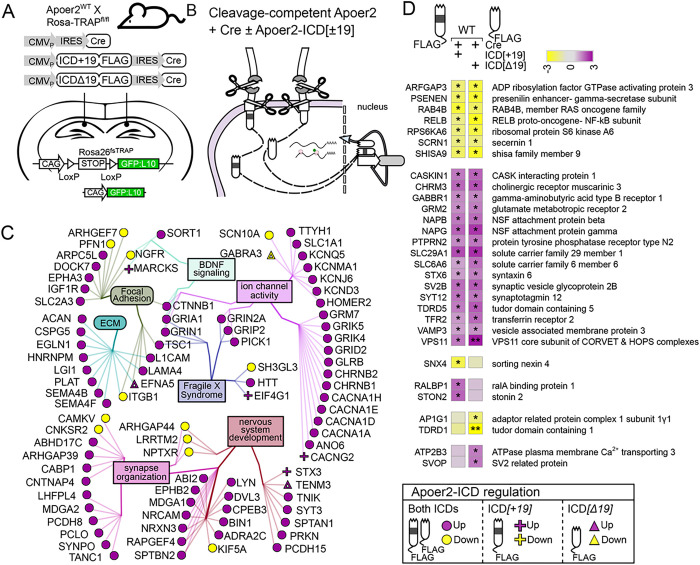
Synaptic effects of overexpressing either Apoer2-ICD in Apoer2^WT^. (**A-B**) Schematic representation of the experiment. (**C**) Diagram depicting the up- (purple symbols) and down- (yellow symbols) regulated transcripts by both ICDs in the same direction (circles), ICD[+19] (plus-sign) or ICD[Δ19] (triangles). (**D**) Heatmap of the log_2_FC differentially-transcribing transcripts in Apoer2^WT^ with either Apoer2-ICD from the network in Figure 1F. Heatmap displaying the log_2_FC expression of the synaptic transcripts not represented in the networks in Panels A and B, respectively. *p<0.05, **p<0.01

Across all Apoer2^WT^ conditions overexpressing either Apoer2-ICD, there were 1,034 differentially regulated transcripts (Fig. 3, S3B, Table S1). Compared to the Apoer2^WT^ injected with lentivirus expressing only Cre, the inclusion of one of the Apoer2-ICDs up- and down-regulated 718 and 314 transcripts, respectively. Only 2 transcripts were regulated in opposite directions by the two ICDs with both up-regulated by Apoer2-ICD[+ 19] and down with Apoer2-ICD[Δ19] (DYNLRB2, KBTBD12). Of the up-regulated transcripts, 612 are augmented by both Apoer2-ICDs, 49 by Apoer2-ICD[+ 19], and 57 by Apoer2-ICD[Δ19]. One of the transcripts up-regulated by only the Apoer2-ICD[+ 19] is LRP3, whose expression is increased by Apoer2 and is reduced in the frontal cortex of postmortem AD brains (Cuchillo-Ibanez et al., 2021). The top functional enrichment categories for these up-regulated transcripts were post-synapse, cell junction organization, and cellular component morphogenesis (Figure S4A, Table S6).

Of the 314 down-regulated transcripts, 203 are reduced by both Apoer2-ICDs, 6 by Apoer2-ICD[+ 19], and 105 by Apoer2-ICD[Δ19]. The top functional enrichment categories for these down-regulated transcripts were cell surface receptor signaling pathways involved in cell-cell signaling, nervous system development, and cellular component morphogenesis (Figure S4A, Table S6). Seven of these genes are Alzheimer’s disease-related, six are down-regulated with both Apoer2-ICDs (COX7A2L, FZD2, KIF5A, MAP2K2, PSENEN, RAF1) and one with only the Apoer2-ICD[Δ19] (SLC39A9). Twelve of the down-regulated transcripts are schizophrenia-related with regulation of 10 by both Apoer2-ICDs (ARHGAP18, GSTM1, HAGH, HDAC1, HTR1F, JAG1, NDEL1, NGFR, S100B, TRPM1) and 2 with only the Apoer2-ICD[Δ19] (RASD2, SMAD5).

Approximately 12% of these transcripts altered in the Apoer2^WT^ hippocampus with one or both of the Apoer2-ICD splice variants are annotated in the SynGO database. Most of these are up-regulated with 86 up-regulated by both, 6 by only the Apoer2-ICD[+ 19], and 4 by only the Apoer2-ICD[Δ19]. Of those down-regulated, 19 are down-regulated by both Apoer2-ICDs, only one by Apoer2-ICD[+ 19], and 3 by Apoer2-ICD[Δ19]. Eighty-nine of these SynGO annotated transcripts made it into our minimal synaptic network from Fig. 1F with 70 transcripts up- and 12 down-regulated by both Apoer2-ICDs (Fig. 3C, D). Only 14 transcripts are uniquely regulated by just one of the ICDs (Fig. 3C, D). Thirty of the ICD-regulated synaptic transcripts did not fall into our gene enrichment network (Fig. 3D). Twenty were up-regulated with 16 by both and 2 each by either ICD only. The other 10 SynGO transcripts not represented in the minimum network were down-regulated with 7 by both, 1 by Apoer2-ICD[+ 19], and 2 by Apoer2-ICD[Δ19]. These down-regulated transcripts consisted of 6 integral synaptic vesicle components (SYT12, SV2B, STX6, PTPRN2, VAMP3, SVOP^[Δ19]^).

### Apoer2-ICD regulation in the Apoer2^cKO^

Across all Apoer2 conditional knockout conditions there are 1,475 ribosome-associated transcripts differentially regulated, with 60% regulated by the lack of Apoer2 and 40% a result of expression of exogenous Apoer2-ICD. Compared to Apoer2^WT^ injected with lentivirus expressing Cre-only, the Apoer2^cKO^ injected with Cre has 323 up- and 584 down-regulated genes (907 total) and 146 of which are annotated in SynGO (44 up; 102 down). Of the ribosome loaded transcripts differentially regulated between Apoer2^WT^ and Apoer2^cKO^ hippocampi, ~ 94% are rescued, leaving only 32 transcripts not rescued by one of the Apoer2-ICDs. Of those rescued, 233 of the 323 up-regulated transcripts were rescued by both Apoer2-ICDs (38 synaptic), while 21 and 31 were rescued by only the Apoer2-ICD[+ 19] or [Δ19], respectively (Table S2). The top functional enrichment categories for these up-regulated transcripts were extracellular matrix/focal adhesion and response to growth factor (Figure S4B, Table S7).

Of the down-regulated transcripts, 120 are not rescued by either of the Apoer2-ICDs (top 10: SLITRK4, ENPP2, KDM3A, DRD1, COPG2, RAB3GAP2, CD2AP, PPFIA2, UBR5, ADGRB3). Of the ~ 80% rescued, 271 of the 584 down-regulated transcripts were rescued by both Apoer2-ICDs, while 14 and 175 were rescued by only the Apoer2-ICD[+ 19] or [Δ19], respectively (Table S2). The top functional enrichment categories for these down-regulated transcripts were positive regulation of organelle organization, supramolecular fiber (actin) organization, and small GTPase mediated signal transduction (Figure S4B, Table S7). This demonstrates the sufficiency of the Apoer2-ICD to regulate synaptic ribosome loaded mRNA abundance in the absence of the full-length receptor.

**Figure 4:**
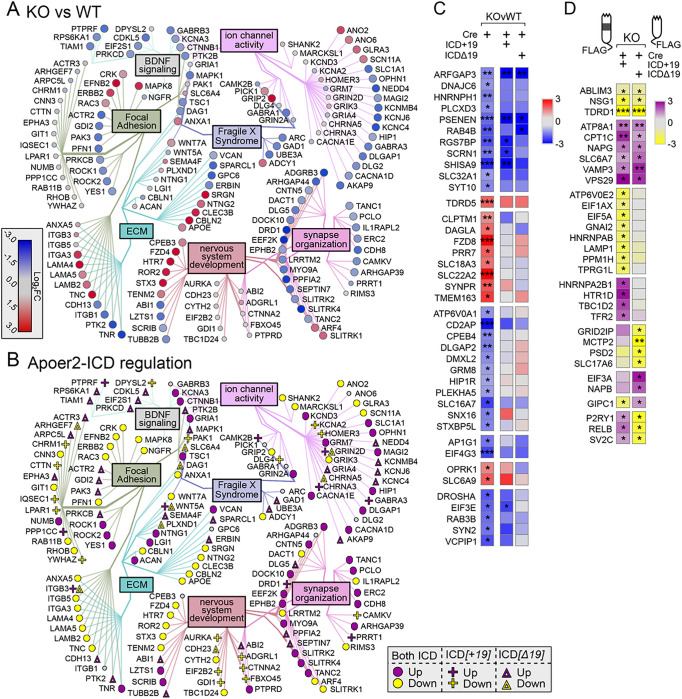
Synaptic effects of Apoer2-ICD in Apoer2^cKO^. (**A-B**) Diagrams depicting the transcripts differentially-translated in the Apoer2^cKO^ at baseline (Cre-only) compared to Apoer2^WT^ (up, red circles; down, blue circles) (**A**) or differentially-translated in Apoer2^cKO^ neurons expressing either ICD[+19] (plus-sign) or ICD[Δ19] (triangles) compared to the baseline Apoer2^cKO^ in the network from Figure 1F (up, purple symbols; down, yellow symbols) (**B**). Note in Panel B, transcripts regulated by both ICDs in the same direction are depicted with circles and those differentially regulated by either the ICD[+19] or ICD[Δ19] are represented by plus-signs or triangles, respectively. (**C-D**) Heatmap displaying the log_2_FC expression of the synaptic transcripts not represented in the networks in Panels A and B, respectively. *p<0.05, **p<0.01

Within our minimal synaptic network from Fig. 1F, there are 164 transcripts regulated overall in the Apoer2^cKO^ conditions (Fig. 4A–B) with 106 transcripts either up- or down-regulated (33 and 73, respectively) compared to the Apoer2^WT^. The other 58 transcripts are regulated by one or both Apoer2-ICD independent of the basal effect of Apoer2-ICD deficiency (genotype-independent). Of those 106 basal ribosome-associated transcripts differentially regulated in the Apoer2^cKO^ compared to the Apoer2^WT^, only 16 down-regulated transcripts are not rescued by either Apoer2-ICD (Fig. 4A–B, Table S2). Of those rescued, all of the up-regulated transcripts were rescued by both Apoer2-ICDs, while half of the down-regulated transcripts were rescued by both Apoer2-ICDs and 3 or 27 by only the Apoer2-ICD[+ 19] or Apoer2-ICD[Δ19], respectively. Gene enrichment revealed up-regulation in focal adhesion and both up- and down-regulation in synapse/nervous system development with increased ribosome association of transcripts coding Wnt-binding proteins (FZD4, FZD8, ROR2) and decreases in transcripts involved in dendrite morphology (ARHGAP44, LZTS1, NEDD4, OPHN1, ROCK2, SEPTIN7, TANC1, UBE3A) (Figure S4B, Table S7).

Of the 54 synaptic ribosome-associated transcripts within the minimal network differentially regulated independent of the basal genotype, both Apoer2-ICDs similarly affected the ribosome association of 24 transcripts with 10 up and 14 down-regulated, while ribosome association of 4 and 6 up-regulated or 16 and 5 down-regulated transcripts were regulated by only the Apoer2-ICD[+ 19] or Apoer2-ICD[Δ19], respectively. Three transcripts are differentially translated in opposite directions by the two Apoer2-ICDs and each are up with Apoer2-ICD[+ 19] and down with the Apoer2-ICD[Δ19] (GRIN2D, ITGB3, P2RY1) (Fig. 4B).

### Synaptic effect of cleavage-resistant Apoer2

In neurons lacking Apoer2, synapse number decreases, and those lacking cleavage of Apoer2 have increased synapse number with reduced synaptic function. These cleavage-resistant variants can also impart additional effects depending on the presence or absence of the proline-rich domain. When this ICD domain is absent, all the above-mentioned effects are further exacerbated, while its presence imparts a Reelin-independent increase in long-term potentiation (Wasser et al., 2014). To probe the effects of the ICD variants on synaptic ribosome-asscoiated transcripts *in vivo*, we compared the baseline effects of cleavage-resistant Apoer2 on ribosome-associated transcripts, as well as the effect of Apoer2-ICD reintroduction. As expected, we observed up-regulation of Apoer2 in both Apoer2 KI mice lacking the OLS (~ 4-fold increase) and down-regulation in the conditional Apoer2^cKO^.

### Apoer2-ICD regulation in the Apoer2 ^Δ16+19^

**Figure 5:**
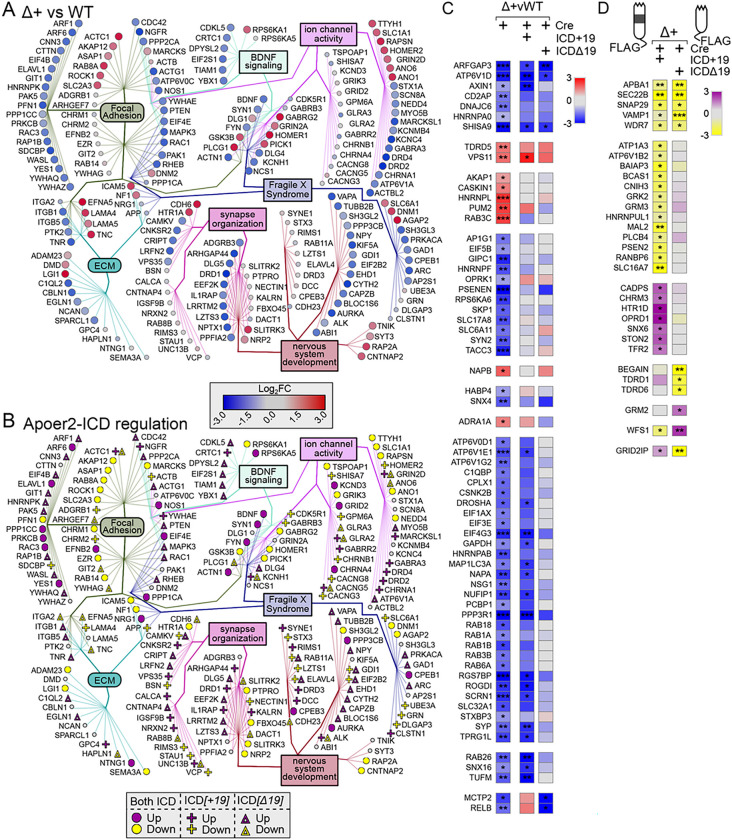
Synaptic effects of Apoer2-ICD in Apoer2 ^Δ16+19^. (**A-B**) Diagrams depicting the transcripts differentially-translated in the Apoer2^Δ16+19^ knockin at baseline (Cre-only) compared to Apoer2^WT^ (up, red circles; down, blue circles) (**A**) or differentially-translated in Apoer2^Δ16+19^ neurons expressing either ICD[+19] (plus-sign) or ICD[Δ19] (triangles) compared to the baseline Apoer2^Δ16+19^ in the network from Figure 1F (up, purple symbols; down, yellow symbols) (**B**). Note in Panel B, transcripts regulated by both ICDs in the same direction are depicted with circles and those differentially regulated by either the ICD[+19] or ICD[Δ19] are represented by plus-signs or triangles, respectively. (**C-D**) Heatmap displaying the log_2_FC expression of the synaptic transcripts not represented in the networks in Panels A and B, respectively. *p<0.05, **p<0.01, ***p<0.001

Across all Apoer2^Δ16+19^ conditions, there are 1,944 ribosome-associated transcripts differentially regulated, with 70% regulated by genotype and 40% a result of expression of exogenous Apoer2-ICD (Fig. 1C–D). Compared to Apoer2^WT^, the Apoer2^Δ16+19^ injected with Cre alone has 617 up- and 735 down-regulated genes (1352 total, Table S3), and the ribosomal-association of the majority of these were normalized by one or both Apoer2-ICDs (85 and 75%, respectively). Of the up-regulated transcripts, 95 are not rescued by either of the Apoer2-ICDs (including Apoer2, shown as LRP8 in Fig. 5) with 9 annotated in SynGO. Of the 522 up-regulated transcripts rescued, 361 were rescued by both Apoer2-ICDs, while 96 and 51 were rescued by only the Apoer2-ICD[+ 19] (synaptic transcripts : ROCK1, NAPB) or [Δ19] (synaptic transcripts: ACTC1, ADRA1A, CDH6, EFNA5, GRIN2D, PLCG1, PRKCD, PUM2), respectively (Table S3). The top functional enrichment categories for these up-regulated transcripts were cell junction organization, head development, and signaling by receptor tyrosine kinases (Figure S4C, Table S8).

Of the down-regulated transcripts, 551 (~ 75%) are rescued by either Apoer2-ICD. 152 of the 735 down-regulated transcripts were rescued by both Apoer2-ICDs, while 52 and 347 were rescued by only the Apoer2-ICD[+ 19] or [Δ19], respectively (Table S3). The top functional enrichment categories for these down-regulated transcripts were synaptic signaling, cell cycle, and post-synapse (Figure S4C, Table S8).

Within our minimal synaptic network from Fig. 1F, there are 211 transcripts regulated overall in the Apoer2^Δ16+19^ conditions (Fig. 5A–D). 146 transcripts are either up- or down-regulated (103 and 43, respectively), and all except 7 of the up- and 25 of the down-regulated transcripts are rescued by reintroduction of the Apoer2-ICD. Within this group, we observed increased ribosomal-association of transcripts modulating synaptic transmission (AKAP12, DNM1, GRIN2D, GSK3B, HOMER1, HTR1A, LGI1, NF1, PICK1, PLCG1, RAB8A, RAPSN) and decreased ribosomal-association transcripts coding for regulators of translation (CPEB1, EEF2K, EIF2B2, EIF2S1, EIF4E) as well as memory impairment (BDNF, DPYSL2, DRD1, DRD2, FYN, NGFR, NOS1, PTEN, SYN1, VPS35). Of the 114 rescued, 41 were rescued by both Apoer2-ICDs, 18 by only the Apoer2-ICD[+ 19], and 55 by only the Apoer2-ICD[Δ19]. The up-regulated transcripts fall mostly under the Fragile X Syndrome category. We observed a general down-regulation in focal adhesion transcripts, 7 of which are involved in integrin signaling (ITGB5, ACTG1, ACTN1, CDC42, FYN, MAPK3, RAC1, RAP1B), as well as synapse/neuron organization including cytoskeleton-binding proteins (ACTN1, ARC, DLG5, DPYSL2, FYN, HNRNPK, KIF5A, SDCBP, SYN1, VAPA).

Of the 65 other ribosomal-associated transcripts within the minimal synaptic network affected by the Apoer2-ICDs independent of their baseline differences to Apoer2^WT^, both ICDs up-regulate 9 and down-regulate 8 transcripts. The Apoer2-ICD[+ 19] and Apoer2-ICD[Δ19] independently regulate 35 and 16 transcripts, respectively. Five transcripts are differentially translated in opposite directions by the two Apoer2-ICDs, 3 are up with Apoer2-ICD[+ 19] and down with the Apoer2-ICD[Δ19] (CACNG5, GLRA2, UNC13B), while ELAVL4 and RAB11A are down with Apoer2-ICD[+ 19] and up with the Apoer2-ICD[Δ19] (Table S3).

### Apoer2-ICD regulation in the Apoer2 ^Δ16Δ19^

Across all Apoer2 ^Δ16Δ19^ conditions, there are 2317 ribosome-associated transcripts differentially regulated. Compared to Apoer2^WT^, the Apoer2^Δ16Δ19^ mice injected with Cre have 582 up- and 872 down-regulated genes (1454 total, Table S4). Of the up-regulated transcripts, only 55 are not rescued by one of the Apoer2-ICDs. Of the 91% rescued, 373 of the 582 up-regulated transcripts were rescued by both Apoer2-ICDs, while 34 and 120 were rescued by only the Apoer2-ICD[+ 19] or [Δ19], respectively (Table S4). The top functional enrichment categories for these up-regulated transcripts were post-synapse, cellular component morphogenesis, and axon (Figure S4D, Table S9).

Of the down-regulated transcripts, 667 are rescued by one of the Apoer2-ICDs Of the 76.5% rescued, 294 of the 872 down-regulated transcripts were rescued by both Apoer2-ICDs, while 36 and 377 were rescued by only the Apoer2-ICD[+ 19] or [Δ19], respectively (Table S4). The top functional enrichment categories for these transcripts were post-synapse, Rho GTPases signaling, and cellular component morphogenesis (Figure S4D, Table S9).

**Figure 6:**
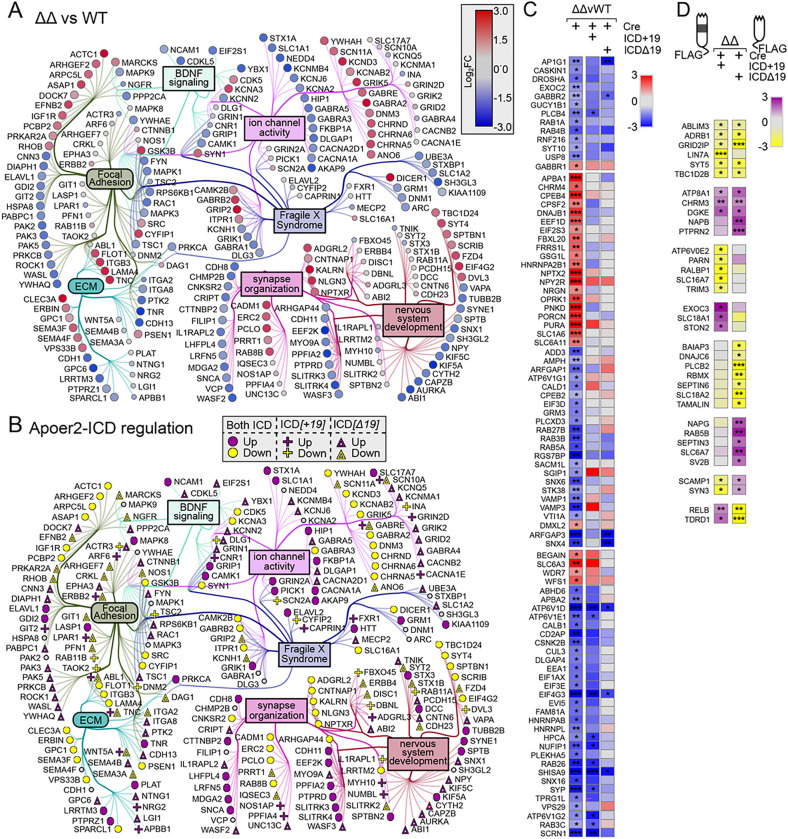
Synaptic effects of Apoer2-ICD in Apoer2^Δ16Δ19^. (**A-B**) Diagrams depicting the transcripts differentially-translated in the Apoer2^Δ16Δ19^ knockin at baseline (Cre-only) compared to Apoer2^WT^ (up, red circles; down, blue circles) (**A**) or differentially-translated in Apoer2^Δ16Δ19^ neurons expressing either ICD[+19] (plus-sign) or ICD[Δ19] (triangles) compared to the baseline Apoer2^Δ16Δ19^ in the network from Figure 1F (up, purple symbols; down, yellow symbols) (**B**). Note in Panel B, transcripts regulated by both ICDs in the same direction are depicted with circles and those differentially regulated by either the ICD[+19] or ICD[Δ19] are represented by plus-signs or triangles, respectively. (**C-D**) Heatmap displaying the log_2_FC expression of the synaptic transcripts not represented in the networks in Panels A and B, respectively. *p<0.05, **p<0.01, ***p<0.001

Within our minimal synaptic network from Fig. 1F, there are 241 SynGO transcripts regulated overall in the Apoer2^Δ16Δ19^ conditions (Fig. 6). One hundred and sixty-three transcripts are either up- or down-regulated (101 and 62, respectively), and all except 2 and 24 of the up- and down-regulated transcripts, respectively, are rescued by reintroduction of the Apoer2-ICD. Of the 172 rescued, 82 were rescued by both Apoer2-ICDs, 2 by only the Apoer2-ICD[+ 19], and 53 by only the Apoer2-ICD[Δ19].

Of the 78 other transcripts within the minimal synaptic network which are affected by the Apoer2-ICDs independent of their baseline differences to Apoer2^WT^, both ICDs up-regulate 11 and down-regulate 2 transcripts. The Apoer2-ICD[+ 19] and Apoer2-ICD[Δ19] independently regulate 24 and 34 transcripts, respectively. Five transcripts are differentially regulated in opposite directions by the two Apoer2-ICDs, 5 are up with Apoer2-ICD[+ 19] and 5 are down with the Apoer2-ICD[Δ19] (ABL1, ERBB2, LPAR1, SCN10A, WNT5A), while ACTR3 and DLG1 are down with Apoer2-ICD[+ 19] and up with the Apoer2-ICD[Δ19] (Fig. 6A–B, Table S4).

### Apoer2-ICD regulation of key AD risk genes and members of the Reelin signaling pathway

**Figure 7:**
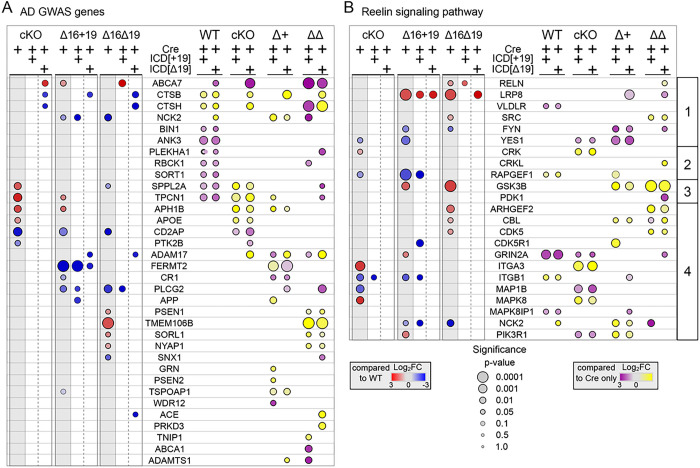
Apoer2-ICD regulation of AD GWAS and Reelin signaling transcripts. Heatmaps depicting the log_2_FC of ribosome-associated AD GWAS (A) or Reelin signaling pathway (B) transcripts between the Apoer2cKO/KI neurons expressing only Cre or Cre with either Apoer2-ICD compared to Apoer2WT expressing only Cre (left panels of A,B) or between neurons expressing either ICD[+19] or ICD[Δ19] compared to the Cre-only translation within each genotype (right panels of A,B). (B) Reelin pathway transcripts are sorted into four groups: the core Reelin receptor complex and associated tyrosine kinases (1), the signaling pathway regulating cadherin trafficking (2), the signaling pathway regulating tau (MAP1B) phosphorylation (3), and the other members of the canonical Reelin pathway (4). Significance is represented by the size of the node.

The inclusion of exon 19 in the Apoer2-ICD is protective against AD pathogenesis, so we next assessed the effect of the Apoer2-ICD splice variants on the regulation of ribosome-associated transcripts of key AD risk genes (Hinrich et al., 2016). To date, there are ~ 90 known AD genetic risk loci with more than 100 potential risk genes (Bellenguez et al., 2022; Kamboh, 2022; Wightman et al., 2021). Of these, 34 transcripts are differentially translated in one or more experimental conditions compared to Apoer2^WT^ (Fig. 7A). Over 50% of these play a critical role in APP/Aβ metabolism, including APP itself, as well as three gamma-secretase subunits (PSEN1, PSEN2 and APH1B). Ten of these are involved in Aβ toxicity or clearance (ABCA7, ACE, BIN1, CD2AP, CR1, CTSB, CTSH, GRN, PTK2B and SORL1), and nine play a role in tau toxicity. Other functional roles of these AD risk genes include regulation of immune response, endocytosis, cytoskeleton, neuron projection, and synaptic function. In Apoer2^cKO^ and cleavage-deficient lines, only CD2AP is significantly regulated in the same direction (down). If we consider transcripts that are *significantly* altered in at least one Apoer2^cKO^ or cleavage-deficient line, we find four with similar down-regulation (log2FC <= −0.5): CR1, FERMT2, PLCG2 and PTK2B. There are no up-regulated AD risk transcripts significantly or similarly up-regulated in all three deficient lines.

A recent genetic linkage analysis implicates the Reelin signaling pathway in AD pathogenesis (Bracher-Smith et al., 2022). When we assess the effect of the Apoer2-ICD across all conditions on the ribosome-association transcripts of these members of the Reelin signaling pathway, nearly half of the core Reelin signaling pathway are altered (Fig. 7B). When we look for similar baseline transcriptional changes in the Apoer2 KI/cKO compared to Apoer2^WT^, the only common regulation is up-regulation of RELN. This is not surprising considering all Apoer2 KI/cKO mice lack the Apoer2-ICD, which we and others have shown to down-regulate RELN expression (Figure S1) (Balmaceda et al., 2014). When we look for commonalties between the two Apoer2 KI lines, we observe similar changes in the core receptor signaling pathway (Fig. 7B) with the expected up-regulation of LRP8 along with up-regulation of GSK3B and CBL and down-regulation of FYN. As for dissimilarities between the two Apoer2 KI lines, we observe opposite regulation of CDK5, ITGB1, and YES1 ribosome-associated transcripts with each down- and up-regulated in Apoer2^Δ16+19^ and Apoer2^Δ16Δ19^, respectively. Between the Apoer2^cKO^ and Apoer2^Δ16+19^, each have similar down-regulated ribosome-associated transcripts of ITGB1, RAPGEF1, and YES1 and up-regulation of ITGA3. Alternatively, ribosome association of GRIN2A and PIK3R1 transcripts is up-regulated in Apoer2^Δ16+19^ and down-regulated in Apoer2^cKO^. The only shared similarity unique to the Apoer2^Δ16Δ19^ and Apoer2^cKO^ cells is up-regulation of CRK ribosome-associated transcripts. None of the transcripts are regulated in only the Apoer2^Δ16+19^, while MAPK8 is up-regulated in Apoer2 ^cKO^ and ARHGEF2 and SRC are up-regulated in Apoer2 ^Δ16Δ19^ alone. Overall, this analysis demonstrates several AD risk genes are regulated either by Apoer2 and/or by the Apoer2-ICD, implicating Apoer2 and its alternative splicing in AD pathogenesis and suggesting the Apoer2/Reelin is a rational target for possible AD therapies.

## Discussion

Apoer2 signaling is critical for brain development, synapse maturation and maintenance as well as function, and inclusion of exon 19 is protective against AD (Beffert et al., 2005; Dumanis et al., 2011; Forster et al., 2010; Niu et al., 2008; Qiu and Weeber, 2007; Strasser et al., 2004; Wasser et al., 2014). Recently, a Reelin gain-of-function mutation has been reported to be protective against early-onset AD (Lopera et al., 2023), thus directly implicating the Reelin/Apoer2 pathway in AD pathogenesis. We know that the abundance of Apoer2 can proportionately regulate synapse number (Dumanis et al., 2011; Wasser et al., 2014), and its cleavage can block extracellular signaling as well as transcription by the release of the ICD (Balmaceda et al., 2014; Koch et al., 2002; Telese et al., 2015).

As transcription does not always reflect ribosome-associated transcripts, we sought to uncover how the Apoer2-ICD regulates the synaptic translatome using a multi-model approach with three genetic Apoer2 mouse lines – each lacking the Apoer2-ICD. Here we demonstrate a large network of differentially regulated ribosome-associated transcripts when the Apoer2-ICD is either not present or not released. This network comprises 15% of all the annotated mouse genes. Of these, half were significantly altered by just the loss of the Apoer2-ICD release and the majority were rescued by reintroducing either Apoer2-ICD with or without the alternatively spliced exon 19. Without the Apoer2-ICD, approximately 30% of the differentially regulated ribosome-associated transcripts were similarly altered across all Apoer2^cKO^ and cleavage-deficient mouse lines – mice with very different phenotypes – suggesting a common Apoer2-ICD regulatory module. From cell adhesion to synaptic scaffolds and the core machinery of chemical neurotransmission, the Apoer2 translatome spans almost all aspects of the synapse and intersects with many brain disorders (AD, schizophrenia, bipolar disorder, autism, epilepsy, and depression) unveiling a novel synaptic role for Apoer2.

Part of this module is enriched in integrins, collagens, laminins, and a variety of cadherin- and cytoskeleton-binding proteins (Fig. 2), which are key players in focal adhesion. Focal adhesions link the extracellular matrix (ECM) to the inside of the cell through complex intracellular interactions. Part of the ECM is suggested to mark the location of eliminated synapses (Jaudon et al., 2021). Considering that neurons of young Apoer2 knockout mice have fewer synapses along with our observation of enhanced ribosome association of ECM transcripts in the Apoer2^cKO^ and the lack of ECM-specific enrichment in any other condition, it is possible these transcripts could truly act as placeholders for the synapses lost upon deletion of Apoer2 (Dumanis et al., 2011).

Reelin, is known to interact with not only NMDA receptors but also integrins and other critical down-stream signaling pathways including those regulating the cytoskeleton (D’Arcangelo et al., 1995). When Reelin’s protein abundance is reduced in neurons, overall ribosome-associated transcript numbers are also reduced. This includes the regulation of the critical regulator of synaptic homeostasis, translation, and cytoskeletal dynamics - activity-regulated cytoskeletal protein (ARC). Reelin signaling leads to the release of the Apoer2-ICD, and in this study, we observed down-regulation of ARC in the translatomes of all ApoER2-ICD cKO/KI genotypes, further implicating the Reelin/Apoer2 pathway in ARC signaling. Likewise, Reelin, in conjunction with integrins, enhances ARC translation in an mTOR-dependent manner (Dong et al., 2003). We observed decreased ribosome associated transcripts of several translation regulators across all genotypes; however, whether these effects are a product of overall reduced Reelin function or a key component of how the lack of the Apoer2-ICD impacts protein translation/mRNA transcription could not be deduced.

### Translatome differences between Apoer2^cKO^ and cleavage-deficient mouse lines: Clues to phenotypic differences

Understanding the subtle differences between the Apoer2^cKO^ and cleavage-deficient mouse models could be the key to unlocking unknown functions of Apoer2 at the synapse. The hippocampal neurons in the uncleavable Apoer2 lines have reduced synaptic transmission despite increased synapse number. The additional loss of exon 19 further increases synaptic density and suppresses synaptic function, all of which are rescued by reducing Apoer2 protein abundance (Wasser et al., 2014). Between the uncleavable Apoer2 lines (Apoer2^Δ16±19^), 15 synaptic transcripts have similar changes in ribosome-associated transcripts compared to Apoer2^WT^ (up: ACTC1, ANO6, ASAP1, GSK3B, LAMA4, LRP8, MARCKS, TNC; down: ATP6V1D, ATP6V1E1, AURKA, EEF2K, EIF4G3, ELAVL1, KIF5A, RGS7BP, SCRN1, SHISA9, SYP) and 5 were significantly regulated in opposite directions (SYT1, SLC6A11 and HNRNPL, ROCK1, SLITRK3). Of these, six regulate translation or mRNA splicing.

Ribosomal-associated transcripts of ROCK1 and SLITRK3 are up-regulated in the Apoer2^Δ16+19^ mouse line and down in the Apoer2 ^Δ16Δ19^ mouse line, while SYT1 and SLC6A11 are down-regulated in the Apoer2^Δ16+19^ mouse line and up in the Apoer2 ^Δ16Δ19^ mouse line. SLITRK3 promotes inhibitory synapse formation and SLC6A11 is a GABA transporter, suggesting GABAergic synaptic input may be elevated in the hippocampi of Apoer2^Δ16+19^ mice and reduced in Apoer2 ^Δ16Δ19^ mice (Li et al., 2017; Takahashi et al., 2012; Yim et al., 2013). This suggests a potential difference in GABAergic input between the uncleavable Apoer2 KI hippocampal neurons, possibly acting to compensate for enhanced LTP in the Apoer2^Δ16+19^ or even compensating for loss of Reelin influence in the Apoer2 ^Δ16Δ19^ neurons (Li et al., 2017; Takahashi et al., 2012; Yim et al., 2013).

Degradation of the ECM enhances the formation of immature filopodial spines with impaired potentiation (Dembitskaya et al., 2021), a deficit reversed by inhibiting small conductance Ca^2+^-activated K^+^-channels in a ROCK1-dependent manner. ROCK1 is a Rho-associated protein kinase required for cytoskeleton remodeling (Nakayama et al., 2000). Within the minimal synaptic network, ROCK1 was the singular ribosome-associated transcript significantly altered in Apoer2^cKO^ and cleavage-deficient mice; however, it was only up-regulated in the Apoer2 ^Δ16+19^ mouse line – the line with enhanced LTP without stimulation by Reelin. This could suggest upregulation of Apoer2 exon 19 specifically at the synapse induces ROCK1 translation leading to increased LTP. We also found reduced ribosome-association of KCNN2 transcripts, a small conductance Ca^2+^-activated K^+^-channel, in the Apoer2^cKO^ and the Apoer2^Δ16Δ19^ lines only. KCNN2 reduces excitability leading to reduced LTP. Interestingly, synaptic activity activates the ubiquitin ligase, UBE3A, leading to KCNN2 ubiquitination (Sun et al., 2015). UBE3A is also down-regulated in the Apoer2^cKO^ and the Apoer2 ^Δ16Δ19^ lines only. When potentiating input enters the synapse, UBE3A ubiquitinates KCNN2 thus reducing its hyperpolarizing input. Both are down-regulated in the Apoer2^cKO^ and Apoer2 ^Δ16Δ19^, while ROCK1 ribosome-associated transcripts are elevated in only Apoer2 ^Δ16+19^. Interestingly, loss of UBE3A is the causative factor in Angelman’s syndrome, a neurodevelopmental disorder resulting in mental retardation and coordination (Madaan and Mendez, 2021).

### Altered ribosome-associated transcripts provide insight into synaptic dysfunction in Apoer2 KI models

While transcripts associated with overarching pathways (BDNF signaling, ion channel activity, focal adhesion, ECM, synapse organization, nervous system development, and Fragile X Syndrome) have already been discussed, it is key to understand how several of these transcript changes may alter synaptic transmission and plasticity. For this discussion, we will only include transcripts that were significantly dysregulated in at least one genotype and rescued with both Apoer2-ICDs, thus demonstrating sufficiency of the Apoer2-ICD to regulate these ribosome-associated transcripts. In Apoer2 ^Δ16+19^ cells, expression of *Prkcb* is decreased. Pkcrb has been shown to increase the abundance of the readily releasable pool, increasing baseline synaptic transmission (Chu et al. 2014). This correlates with Apoer2^Δ16+19^ demonstrating decreased hippocampal output when compared to Apoer2 WT mice (Wasser et al., 2014). Further, Apoer2^Δ16+19^ mice have decreased levels of ribosome-associated *Slc17a3* transcripts. *Slc17a3* codes for VGlut3, and VGlut3 KO mice demonstrated decreased mIPSC frequency and an increased threshold for LTD (Fasano et al., 2017). Apoer2^Δ16+19^ mice also demonstrated decreased ribosome-associated transcripts of both *Syn1* and *Syn2*. Knockout of either synapsin gene leads to decreased post-tetanic potentiation, a form of short term-potentiation, further demonstrating Apoer2-dependent dysregulation of synaptic transmission and plasticity (Valente et al., 2012; Rosahl et al. 1995). Meanwhile, ribosome-associated *Syn1* levels are increased in Apoer2^Δ16Δ19^ mice, demonstrating a possible rationale for the phenotypic variation between Apoer2 KI mouse models. Ribosome-associated transcripts of both *Lgi1* and *Adam23* are increased in Apoer2^Δ16+19^ mice. Lgi1 and possibly Adam23 work to modulate Kv1.1 levels and subsequently neuronal excitability while Lgi1 KO neurons also demonstrated decreased AMPA receptor currents (Fels et al., 2021). Lastly, both Apoer2 KI models demonstrated increased levels of either ribosome-associated *Rab8a* or *Rab8b*. The protein products of these transcripts are both implicated in transporting GluA1 subunits to post-synaptic spines during both receptor homeostasis and LTP (Gerges et al., 2004). This is not an exhaustive list of all synaptic genes dysregulated in cleavage resistant Apoer2 KI models, but it demonstrates how altered ribosome-associated transcripts in Apoer2^Δ16±19^ mice cover a vast swath of synaptic functions including membrane potential homeostasis (*Lgi1* and *Adam23*), baseline synaptic transmission (*Pkcrb* and *Slc17a3*), short term synaptic plasticity (*Syn1* and *Syn2*), LTD (*Slc17a3*), and LTP (*Rab8*). Further, by demonstrating how reintroduction of the soluble Apoer2-ICD rescues all of these transcripts, we highlight the importance of Apoer2-ICD cleavage on synaptic transmission and plasticity.

### Disease Implications

Our data reveal Apoer2 as a potent modulator of AD risk genes (Fig. 7A). The majority of these are known to regulate APP processing and Aβ/tau pathology. The Apoer2-ICD[+ 19] specifically down-regulated ribosomal association of APP transcripts in the Apoer2^Δ16+19^ hippocampi. In Apoer2^cKO^ and cleavage-deficient lines, at least one member of the gamma-secretase complex was also down-regulated after expression of one of the Apoer2-ICDs. In Apoer2^cKO^ and cleavage-deficient lines, the cytoskeletal regulator CD2AP is down-regulated, and the Apoer2-ICD primarily rescues CD2AP levels in the Apoer2^cKO^ mice (and to a lesser extent in the Apoer2^Δ16Δ19^ mice). CD2AP overexpression shunts APP from early endosomes towards lysosomal degradation (Furusaway et al., 2019). The AD risk gene FERMT2 also regulates APP whereby silencing or overexpression of FERMT2 results in increased or decreased surface levels of mature APP, leading to respective changes in secreted amyloid-β (Capuis et al., 2017). In our analysis, only Apoer2^Δ16+19^ mice demonstrated decreased ribosome-associated Fermt2 transcripts, an effect partially corrected by addition of the Apoer2-ICD[Δ19]. Together, these findings suggest Apoer2 and its alternative splicing influences APP homeostasis through several independent mechanisms.

Another AD risk gene, PTK2B, which encodes the Pyk2 protein, is also down-regulated in all Apoer2-ICD lacking mouse lines. Pyk2 has been implicated in the synaptic toxicity of amyloid-β oligomers in mice as well as suppressing tau toxicity in *Drosophila* (Douerlen et al., 2017 and Salazar et al., 2019). Finally, we also found that overexpression of either Apoer2-ICD in Apoer2^WT^ mice increased the ribosome-associated transcripts of the homolog of the AD risk gene BIN1. Decreased expression of BIN1 led to suppression of Tau-mediated neurotoxicity (Chapuis et al., 2013). This suggests Apoer2 and its ICD indirectly influence both amyloid β and tau toxicity by altering the ribosome-associated transcripts of known amyloid β and tau interactors.

Alongside AD, Reelin is implicated in several neuropsychiatric and neurodegenerative diseases, such as autism, schizophrenia, bipolar disorder, and major depression (reviewed in (Caruncho et al., 2004; Costa et al., 2001; D’Arcangelo, 2006; Folsom and Fatemi, 2013; Knuesel, 2010; Lakatosova and Ostatnikova, 2012; Lane-Donovan et al., 2015)). Despite different pathologies, reduced Reelin function is a common factor in each disorder (Guidotti et al., 2000; Herring et al., 2012; Impagnatiello et al., 1998). With the lack or overexpression of the Apoer2-ICD, we find a similar disease imprint as its ligand, Reelin. One key transcript that presents in our dataset, GSK3β, is a central hub in AD and schizophrenia. GSK3β is upregulated in both cleavage-resistant Apoer2 mouse models, an effect rescued by either Apoer2-ICD (Figs. 5, 6). Reelin signaling through Apoer2 and Vldlr inhibits GSK3β, which when active, phosphorylates tau (Jossin and Goffinet, 2007). This inhibition prevents hyperphosphorylation of tau, an event known to result in tau aggregation and formation of the hallmark AD neurofibrillary tangles. This suggests Reelin not only reduces the activity of GSK3β by signaling through Apoer2, but also by reducing the number of ribosome-associated transcripts of this constitutively active kinase via ligand-binding induced cleavage of Apoer2.

Another synaptic hub protein that is implicated in AD and schizophrenia, β-catenin (CTNNB1), is a core participant in Wnt-signaling, and the number of ribosome-associated CTNNB1 transcripts is down-regulated in the Apoer2^cKO^ (Fig. 4). The Wnt-signaling pathway, much like Reelin signaling, regulates brain development, synapse formation and synapse function (Inestrosa et al., 2012). Two other members of the low-density lipoprotein receptor family, LRP5 and LRP6, are central to Wnt signaling. Both are co-receptors with the Frizzled receptor (FZD) for Wnt ligands, resulting in reduced degradation of β-catenin (Nusse and Clevers, 2017; Pohlkamp et al., 2017). Additionally, neuronal knockout of Lrp6 in mice causes deficits in synaptic integrity and memory formation. When crossed with the APP/PS1 line, Lrp6 mice demonstrated enhanced Aβ production leading to inhibition of Wnt signaling, a finding also seen in Alzheimer brains (Liu et al., 2014). Interestingly, clozapine reduced β-catenin and TCF-4 (encoded by TCF7L2), an effect mediated through down-regulation of TNIK (Traf2- and Nck-interacting kinase) (Yuan et al., 2021). TNIK is up-regulated by either Apoer2-ICD (Fig. 3), which provides another mechanism by which reduced Reelin signaling could impart risk for psychosis. These experiments reveal a landscape of critical synaptic proteins altered in the absence of the Apoer2-ICD. Further exploration is required to understand how these changes affect the synaptic environment; however, our study, together with the protective nature of a Reelin gain-of-function mutation (Lopera et al., 2023) demonstrates the importance of Reelin/Apoer2 signaling for AD pathogenesis.

### Limitations of the study

A major limitation of this study is the inability to quantify the proteomic consequences of the Apoer2-ICD. Because we are reintroducing the Apoer2-ICD through viral infection, only a subset of cells (~ 10%) will express our construct. Therefore, the signal-to-noise ratio of bulk RNA-Seq or proteomics would be too low to ascertain Apoer2-ICD-dependent changes *in vivo*. For this reason, we utilized our Cre-dependent TRAP protocol to isolate mRNA strictly from lentivirus-infected - and thus ICD expressing - cells. While this approach cannot reveal differential protein expression in the infected cells, the specificity and sensitivity of the technique allowed us to detect changes of ribosome-primed transcripts secondary to the presence or absence of Apoer2-ICD.

Further, while our approach does not specifically differentiate between hippocampal cell-types, it does reflect the physiological context of the hippocampus where Apoer2 is expressed in all cells. By complementing our data analysis with single cell sequencing databases, we were nonetheless able to ascribe the majority of the mRNA expression changes to neurons (Karlsson et al., 2021) (Figure S5), demonstrating the effect of Apoer2-ICD expression primarily on hippocampal neurons.

## Data Availability

Primary data have been uploaded to the following address. https://dataverse.tdl.org/dataverse/Apoer2-ICD_translatome. Further information and requests for resources, reagents, and data should be directed to and will be fulfilled by the lead contact: Dr. Catherine Wasser. (catherine.wasser@utsouthwestern.edu).

## Abbreviations

ABCA7: ATP Binding Cassette Subfamily A Member 7
ABI2: Abl Interactor 2
ABL1: ABL proto-oncogene 1
ACE: Angiotensin I Converting Enzyme
ACTA2: Actin Alpha 2, Smooth Muscle
ACTC1: Actin Alpha Cardiac Muscle 1
ACTG1: Actin Gamma 1
ACTN1: Actinin Alpha 1
ACTR3: Actin Related Protein 3
AD: Alzheimer’s disease
ADAM23: ADAM Metallopeptidase Domain 23
ADGRB3: Adhesion G Protein-Coupled Receptor B3
ADRA1A: Adrenoceptor Alpha 1A
AKAP12: A-Kinase Anchoring Protein 12
AMPA: α-amino-3-hydroxy-5-methyl-4-isoxazolepropionic acid
ANO6: Anoctamin 6
AP1G1: AP-1 complex subunit gamma-1
APH1B: Aph-1 Homolog B, Gamma-Secretase Subunit
APOE: Apolipoprotein E
APOER2: Apolipoprotein E receptor 2
APP: Amyloid precursor protein
ARC: Activity Regulated Cytoskeleton Associated Protein
ARHGAP18: Rho GTPase Activating Protein 18
ARHGAP44: Rho GTPase Activating Protein 44
ARHGEF2: Rho/Rac Guanine Nucleotide Exchange Factor 2
ARID1B: AT-Rich Interaction Domain 1B
ASAP1: ArfGAP With SH3 Domain, Ankyrin Repeat And PH Domain 1
ATP6V1D: ATPase H + Transporting V1 Subunit D
ATP6V1E1: ATPase H + Transporting V1 Subunit E1
AURKA: Aurora Kinase A
Aβ: Amyloid beta
BDNF: Brain-derived neurotrophic factor
BIN1: Bridging Integrator 1
BRWD1: Bromodomain And WD Repeat Domain Containing 1
CACNG5: Calcium Voltage-Gated Channel Auxiliary Subunit Gamma 5
CASP3: Caspase 3
CBL: CBL Proto-Oncogene
CD2AP: CD2 Associated Protein
CDC42: Cell Division Cycle 42
CDH6: Cadherin 6
CDK5: Cyclin-dependent kinase 5
CDKL5: cyclin-dependent kinase-like 5
COL1A2: Collagen Type I Alpha 2 Chain
COPG2: COPI Coat Complex Subunit Gamma 2
COX7A2L: Cytochrome C Oxidase Subunit 7A2 Like
CPEB1: Cytoplasmic polyadenylation element-binding protein 1
CR1: Complement C3b/C4b Receptor 1 (Knops Blood Group)
CRK: CRK Proto-Oncogene, Adaptor Protein
CTNNB1: Catenin Beta 1
CTSB: Cathepsin B
CTSH: Cathepsin H
DBYSL2: Dihydropyrimidinase Like 2
DLG1: Discs Large MAGUK Scaffold Protein 1
DLG5: Discs Large MAGUK Scaffold Protein 5
DNM1: Dynamin 1
DPYSL2: Dihydropyrimidinase Like 2
DRD1: Dopamine Receptor D1
DRD2: Dopamine Receptor D2
DSP: desmoplakin
DYNLRB2: Dynein light chain roadblock-type 2
ECD: Extracellular domain
ECM: Extracellular matrix
EEF2K: Eukaryotic Elongation Factor 2 Kinase
EFNA5: Ephrin A5
EIF2B2: Eukaryotic initiation factor-2B
EIF2S1: Eukaryotic Translation Initiation Factor 2 Subunit Alpha
EIF4E: Eukaryotic translation initiation factor 4E
EIF4G3: Eukaryotic Translation Initiation Factor 4 Gamma 3
ELAVL4: ELAV Like RNA Binding Protein 4
ENPP2: Ectonucleotide Pyrophosphatase/Phosphodiesterase 2
ERBB2: Receptor tyrosine-protein kinase erbB-2
FERMT2: FERM Domain Containing Kindlin 2
FYN: Fyn Proto-Oncogene, Src Family Tyrosine Kinase
FZD: Frizzled
FZD2: Frizzled Class Receptor 2
FZD4: Frizzled Class Receptor 4
FZD8: Frizzled Class Receptor 8
GABA: Gamma-aminobutyric acid
GFP: Green fluorescent protein
GLRA2: Glycine receptor subunit alpha-2
GLUA1: Glutamate Ionotropic Receptor AMPA Type Subunit 1
GRIN2D: Glutamate Ionotropic Receptor NMDA Type Subunit 2D
GRN: Progranulin
GSEA: Gene Set Enrichment Analysis
GSK3B: Glycogen Synthase Kinase 3 Beta
GSTM1: Glutathione S-Transferase Mu 1
HAGH: Hydroxyacylglutathione hydrolase
HDAC1: Histone deacetylase 1
HNRNPK: Heterogeneous Nuclear Ribonucleoprotein K
HNRNPL: Heterogeneous Nuclear Ribonucleoprotein L
HTR1A: 5-Hydroxytryptamine Receptor 1A
HTR1F: 5-Hydroxytryptamine Receptor 1F
ICD: Intracellular domain
IRES: Internal ribosome entry site
ITGA3: Integrin Subunit Alpha 3
ITGB1: Integrin Subunit Beta 1
ITGB3: Integrin Subunit Beta 3
ITGB5: Integrin Subunit Beta 5
JAG1: Jagged Canonical Notch Ligand 1
KBTBD12: Kelch Repeat And BTB Domain Containing 12
KCNN2: Potassium Calcium-Activated Channel Subfamily N Member 2
KDM3A: Lysine Demethylase 3A
KDM4B: Lysine-specific demethylase 4B
KIF5A: Kinesin family member 5A
LAMA4: Laminin Subunit Alpha 4
LGI1: Leucine-rich glioma-inactivated 1
LOAD: Late-onset Alzheimer’s disease
LPAR1: Lysophosphatidic Acid Receptor 1
LRP1: Low-density lipoprotein receptor related protein 1
LRP3: Low-density lipoprotein receptor-related protein 3
LRP5: LDL Receptor Related Protein 5
LRP6: LDL Receptor Related Protein 6
LRP8: Low-density lipoprotein receptor related protein 8
LTP: Long-term potentiation
LZTS1: Leucine Zipper Tumor Suppressor 1
MAP2K2: Mitogen-Activated Protein Kinase Kinase 2
MAPK3: Mitogen-Activated Protein Kinase 3
MAPK8: Mitogen-Activated Protein Kinase 8
MARCKS: Myristoylated alanine-rich C-kinase substrate
mIPSC: Miniature inhibitory postsynaptic currents
mTOR: Mammalian target of rapamycin
NAPB: NSF Attachment Protein Beta
NCOA2: Nuclear Receptor Coactivator 2
ND6: NADH dehydrogenase 6
NDEL1: NudE Neurodevelopment Protein 1 Like 1
NEDD4: NEDD4 E3 Ubiquitin Protein Ligase
NF1: Neurofibromatosis type 1
NGFR: Nerve Growth Factor Receptor
NOS1: Nitric oxide synthase 1
OPHN1: Oligophrenin 1
OPRK1: Opioid Receptor Kappa 1
P2RY1: Purinergic Receptor P2Y1
PI3K: phosphoinositide 3-kinase
PICK1: Protein Interacting With PRKCA 1
PIK3R1: Phosphoinositide-3-Kinase Regulatory Subunit 1
PLCG1: Phospholipase C gamma 1
PLCG2: Phospholipase C Gamma 2
PPFI2A: PTPRF Interacting Protein Alpha 2
PRKCB: Protein kinase C beta type
PRKCD: Protein kinase C delta type
PS1: Presenilin-1
PSD-95: Post-synaptic density protein 95
PSEN2: Presenilin 2
PSENEN: Presenilin Enhancer
PTEN: Phosphatase and tensin homolog
PTK2B: Protein Tyrosine Kinase 2 Beta
PTPRN2: Protein Tyrosine Phosphatase Receptor Type N2
PUM2: Pumilio RNA Binding Family Member 2
PYK2: Protein Tyrosin Kinase 2
RAB11A: RAB11A, Member RAS Oncogene Family
RAB3B: RAB3B, Member RAS Oncogene Family
RAB3GAP2: RAB3 GTPase Activating Non-Catalytic Protein Subunit 2
RAB8A: RAB8A, Member RAS Oncogene Family
RAB8B: RAB8B, Member RAS Oncogene Family
RAC1: Rac Family Small GTPase 1
RALGAPA2: Ral GTPase Activating Protein Catalytic Subunit Alpha 2
RAP1B: RAP1B, Member Of RAS Oncogene Family
RAPGEF1: Rap Guanine Nucleotide Exchange Factor 1
RAPSN: Receptor Associated Protein Of The Synapse
RASAL2: RAS Protein Activator Like 2
RASD2: RASD Family Member 2
RCC2: Regulator Of Chromosome Condensation 2
RGS7BP: Regulator Of G Protein Signaling 7 Binding Protein
ROCK1: Rho associated coiled-coil containing protein kinase 1
ROCK2: Rho Associated Coiled-Coil Containing Protein Kinase 2
ROR2: Receptor Tyrosine Kinase Like Orphan Receptor 2
RPTOR: Regulatory Associated Protein Of MTOR Complex 1
S100B: S100 Calcium Binding Protein B
SCN10A: Sodium Voltage-Gated Channel Alpha Subunit 10
SCRN1: Secernin 1
SDCBP: Syndecan Binding Protein
SETD2: SET domain containing 2
SHISA9: Shisa Family Member 9
SLC17A3: Solute Carrier Family 17 Member 3
SLC1A1: Solute Carrier Family 1 Member 1
SLC39A9: Solute Carrier Family 39 Member 9
SLC6A11: Solute Carrier Family 6 Member 11
SLITRK3: SLIT And NTRK Like Family Member 3
SLITRK4: SLIT And NTRK Like Family Member 4
SMAD5: SMAD Family Member 5
SORL1: Sortilin Related Receptor 1
SRC: SRC Proto-Oncogene, Non-Receptor Tyrosine Kinase
STX6: Syntaxin 6
SV2B: Synaptic vesicle glycoprotein 2B
SVOP: SV2 Related Protein
SYN1: Synapsin I
SYN2: Synapsin 2
SYP: Synaptophysin
SYT12: Synaptotagmin 12
TANC1: tetratricopeptide Repeat, Ankyrin Repeat And Coiled-Coil Containing 1
TCF7L2: Transcription factor 7-like 2 (T-cell specific, HMG-box)
TFAP2B: Transcription Factor AP-2 Beta
TNC: Tenascin C
TNIK: TRAF2 And NCK Interacting Kinase
TRAP: Translating-ribosome affinity purification
TRN: Tenascin R
TRPM1: Transient Receptor Potential Cation Channel Subfamily M Member 1
TUBB2B: Tubulin Beta 2B Class IIb
UBE3A: Ubiquitin Protein Ligase E3A
UBR5: ubiquitin-protein ligase N-recognin 5
UNC13B: Unc-13 Homolog B
VAMP3: Vesicle Associated Membrane Protein 3
VAPA: VAMP Associated Protein A
VGLUT3: Vesicular Glutamate Transporter 3
VLDLR: Very low-density lipoprotein receptor
VPS35: Vacuolar protein sorting ortholog 35
WNT5A: Wnt Family Member 5A
YES1: YES Proto-Oncogene 1, Src Family Tyrosine Kinase
ZNF644: Zinc Finger Protein 644
